# Phylogenetic ubiquity of the effects of altered ubiquinone biosynthesis on survival

**DOI:** 10.18632/aging.100310

**Published:** 2011-03-17

**Authors:** Siegfried Hekimi, Bryan Hughes

**Affiliations:** Department of Biology, McGill University, Québec, Montréal, Canada

The widespread use of unicellular and invertebrate model systems has revealed that the molecular mechanisms underlying cellular functions are exceedingly well conserved. The expanding research into the molecular mechanisms of aging keeps yielding this answer too [1, 2]. A perfect example of this is a study by Gonidakis, Finkel and Longo appearing in the present issue of Aging, which shows that a disruption of the biosynthesis of ubiquinone leads to increased survival of *E. coli* during stationary phase, associated with an increased resistance to treatments that induce oxidative stress [3]. These effects are dependent upon the presence of an intact *arcA* gene, which encodes the regulatory component of the ArcA/ArcB system, a hypoxia-inducible system of transcriptional regulation.

To understand why these findings might be an example of evolutionary conservation a little background is needed. Interventions that disrupt mitochondrial function can increase lifespan in a variety of organisms including yeast [4], *Caenorhabditis elegans*[5-10], Drosophila [11], and mice [12-14]. One type of intervention is the disruption of mitochondrial function through the reduction of the expression of mitochondrial genes by RNAi, which increases lifespan in worms [5, 10] and in flies [11], possibly via a mitochondria-specific stress response [9]. Another type of intervention is a specific alteration of mitochondrial electron transport that alters the generation of reactive oxygen species (ROS) [15, 16]. For example, *C. elegans isp-1* and *nuo-6* mutants, which carry point mutations in subunits of the mitochondrial electron transport chain (ETC) display an elevated generation of mitochondrial superoxide which appears to be causal to their increased lifespan. Indeed, antioxidants suppress the mutants’ longevity and pro-oxidant treatment of the wild type phenocopies it [16]. *clk-1* (called *Mclk1* in mice) is another gene that has been studied in this context. It encodes a mitochondrial hydroxylase that is necessary for the biosynthesis of ubiquinone [6, 17, 18]. Ubiquinone (a.k.a. co-enzyme Q) is an electron transporter and antioxidant that is ubiquitous in the membranes of all organisms [19]. *C. elegans clk-1* mutants [20] and mouse *Mclk1^+/−^* mutants [12, 13] are long-lived and have been shown to have elevated generation of mitochondrial ROS [16, 21]. In cultured vertebrate cells mitochondrial ROS have been shown to help stabilize and thus induce the protective activity of the hypoxia-inducible factor 1α (HIF-1α) [22-24]. It is striking therefore that HIF-1α has been tentatively implicated in the mechanisms of longevity of both *C. elegans clk-1* [25] and mouse *Mclk1*^+/−^ mutants [26].

Of course bacteria have no mitochondria; however, as the evolutionary ancestors of mitochondria, they have a plasma membrane ETC partly homologous to that of the organelle. As in mitochondria, the bacterial ETC appears to produce significant amounts of ROS [27]. Like eukaryotic cells, they have transcription factors sensitive to hypoxia. The ArcA/ArcB two-component system is one of the key pathways up-regulated in response to anaerobic conditions. Although not genetically homologous to eukaryotic HIF-1α, there are interesting parallels between the systems. While activated by the redox state of the bacterial quinone pool rather than by ROS [28-30], Arc activation is required for the resistance of *E. coli* to induced oxidative stress [31]. Thus, the work of Longo and co-workers in *E. coli* suggests that there might be a truly universal link between ubiquinone, ROS generation, hypoxia-sensitive transcription factors and cellular survival.
